# Emergency Department Violence: A Growing Challenge for Healthcare Workers in Saudi Arabia

**DOI:** 10.7759/cureus.52455

**Published:** 2024-01-17

**Authors:** Fadhilah Alobaidan, Mohammed I Al-Bazroun, Hussain Al Aman, Saleh Al Jaroudi, Afnan Al Qattan, Ali Hbiesheh, Husna Al Nassar

**Affiliations:** 1 Nursing, Royal Commission Health Services Program, Jubail, SAU; 2 Nursing Professional Development, Qatif Health Network/Nursing Affairs, Qatif, SAU; 3 Optometry, Maternity and Child Hospital, Qatif, SAU; 4 Nursing, Dammam Health Network, Qatif, SAU

**Keywords:** hospital safety, saudi arabia, emergency departments, healthcare workers, workplace violence

## Abstract

Background

Workplace violence in healthcare settings, particularly in emergency departments (EDs), is a critical issue affecting healthcare providers and the quality of patient care. This study's primary aim was to determine the prevalence and types of workplace violence experienced by healthcare workers in Saudi Arabian EDs, assess the physical and psychological consequences of such violence, and explore the factors contributing to its occurrence.

Methods

Employing a descriptive, cross-sectional research design, the study was conducted in two phases at three hospitals in the Eastern Region of Saudi Arabia. It targeted ED healthcare workers, including physicians, nurses, and support staff. Data were collected via a questionnaire disseminated through social media, addressing incidents of violence, their nature, and subsequent responses.

Results

The findings indicate a significant incidence of workplace violence in EDs, predominantly involving verbal abuse and physical aggression, mostly initiated by patients' companions. The majority of these incidents occurred prior to patient-physician interaction, with nurses frequently being the primary victims. Although incidents were more often formally reported than informally, a substantial number remained unreported. There was no significant correlation between the experienced violence and variables like working hours or hospital governance.

Conclusion

The research highlights the urgent need for effective policies and strategies to mitigate workplace violence in healthcare settings in Saudi Arabia. It emphasizes the necessity of implementing comprehensive violence prevention and intervention programs to ensure a safer working environment for healthcare professionals.

## Introduction

Workplace violence in healthcare settings, particularly emergency departments (EDs), is a multidimensional and universal problem. It encompasses various forms of aggressive and hostile behavior, including physical assault, verbal abuse, threats, and harassment, directed toward healthcare providers, such as doctors, nurses, and support staff [1]. The consequences of workplace violence in healthcare settings can be profound, affecting both the victims and the quality of patient care [2]. The World Health Organization (WHO) defines violence as the intentional use of physical force or power, whether threatened or used, against oneself, another person, a group, or a community, with consequences or a high likelihood of causing harm, death, mental distress, maldevelopment, or deprivation [3].

The work environment in EDs is quite dynamic [4]. Due to the high workload, the nature of care, the unpredictable nature of events with little control over the workflow, the high patient load, and the lengthy working hours, it is known to be a stressful and hectic environment, which increases the risk of exposure to aggression and violence from patients and visitors [5].

Studies from around the world have documented the prevalence of workplace violence against healthcare workers in EDs. Research conducted in the United States [1] and Turkey [6] highlighted the disturbingly high incidence of violence in these settings. Similarly, studies conducted in Hong Kong [7], Australia [8], and various other countries have reported similar concerns.

Within the broader context of healthcare, the WHO recognizes workplace violence as a critical issue that affects the quality of patient care and the well-being of healthcare workers [9]. Additionally, the Bureau of Labor Statistics in the United States reports healthcare and social assistance as one of the industries with a high risk of workplace violence-related injuries and illnesses [10].

In Saudi Arabia, an emerging body of research has begun to examine the prevalence and impact of workplace violence against healthcare workers in EDs. Studies conducted in Abha City [11], Riyadh [12], and other locations [13] have found alarming rates of violence towards healthcare workers, emphasizing the significance of this issue in the country.

Saudi Arabia, with its unique cultural and healthcare context, presents a distinct landscape for examining workplace violence within EDs. The rapid expansion of healthcare facilities and the increasing demand for services have raised concerns about the safety and well-being of healthcare providers.

While existing research provides valuable insights into workplace violence against healthcare workers in Saudi Arabia, there is a need for a comprehensive cross-sectional descriptive study that encompasses the entire spectrum of this issue. This study aims to Fill this gap by assessing the prevalence, types, and consequences of violence experienced by healthcare workers in EDs within the Eastern Province of Saudi Arabia. Moreover, it seeks to explore the factors contributing to workplace violence and gather recommendations for violence prevention and intervention strategies tailored to the Saudi Arabian context.

The insights gained from this study will be invaluable for healthcare institutions, policymakers, and stakeholders in Saudi Arabia. They will aid in the development of evidence-based strategies and policies to address the growing challenge of workplace violence, ultimately ensuring the safety and well-being of healthcare workers and the provision of high-quality patient care.

## Materials and methods

Study design and setting

A descriptive, cross-sectional research approach was used for this research to explore the prevalence of violence experienced by healthcare workers in the ED. The study was divided into two phases. Phase one was focused on Royal Commission Health Services Program (RCHSP) staff currently working in ED, and phase two was conducted and involved another two hospitals in the Eastern Region, in Qatif Health Network (QHN) and Dammam Health Network (DHN), for comparison purposes and to understand the nature of violence and its relation to the location of cities. Phase one took place in July 2023, and phase two took place in the EDs in October 2023.

Study population and sampling

The study's target population encompassed a comprehensive spectrum of ED healthcare workers within the Eastern Region of the Kingdom of Saudi Arabia. This included professionals across various roles: physicians, nurses, nurse assistants, paramedics, emergency medical technicians, and pharmacists. The inclusion of these diverse professional categories within the study's scope is critical for obtaining a holistic understanding of workplace violence across different levels of healthcare provision. 

The study focused on three major healthcare networks in this region: the Royal Commission Hospital (RCHSP) in Jubail, the Qatif Health Network (QHN), and the Dammam Health Network (DHN). This selection of hospitals from distinct health networks enhances the representativeness of the study, potentially capturing variations in workplace violence experiences and policies across different organizational contexts.

A non-probability convenience sampling technique was used to select healthcare workers from various EDs in RCHSP, QHN, and DHN across Saudi Arabia.

Tools and instrumentation

The study utilized a structured questionnaire specifically developed to assess violence experienced by healthcare workers. The questionnaire exhibited robust internal consistency, evidenced by a Cronbach's alpha score ranging between 0.77 and 0.81. This instrument encompassed multiple sections with a total of 32 questions, beginning with demographic queries capturing participants' profiles, including their professional roles, experience, and work shifts. The core of the questionnaire focused on the frequency, types (verbal and physical), and perpetrators of violence experienced or witnessed. It also probed into the circumstances under which violence occurred, such as the phase of patient care and the timing of incidents. Crucially, the questionnaire examined the reporting behaviors of healthcare workers, exploring both formal and informal channels used for reporting violent incidents. Designed with a blend of multiple-choice and Likert-scale questions, this tool was integral in quantifying and understanding the nuances of violence in healthcare settings, providing essential insights for devising targeted interventions.

Data collection and management plan

The questionnaire was administered to emergency department healthcare workers at the RCHSP, QHN, and DHN. It was distributed using an online link, providing respondents with a two-month timeframe for completion. At the end of this period, a total of 157 completed responses were received. The research team carefully designed the data collection form, incorporating subjective content validation reached by consensus. This method ensured the questionnaire thoroughly covered all relevant aspects of the study, enhancing its clarity and comprehensiveness for participants.

Statistical analysis

This cross-sectional study analyzed data on workplace violence against healthcare workers in emergency departments in Saudi Arabia using IBM SPSS Statistics Version 25 (IBM, Inc., Armonk, US). Descriptive statistics, including frequencies and percentages, were employed to summarize sociodemographic characteristics, prevalence, types, and causes of violence. Inferential statistics involved chi-squared tests to explore associations between categorical variables, such as shift lengths and exposure to violence. A p-value of ≤0.05 was adopted for statistical significance across all tests. Results were presented in tables, emphasizing practical implications for preventing and managing workplace violence in healthcare settings. Limitations related to data and methodology were acknowledged, particularly regarding their impact on the generalizability of the findings.

Ethical considerations

The Institutional Review Board (IRB) of Royal Commission Health Facilities granted ethical approval. The approval date was March 10, 2023, and the reference number was IRB-RCH-51. Before filling out the survey, each participant was asked to give their informed consent. Participants in the study were allowed to cancel at any time, and participation was entirely voluntary. Participants were informed that their personal information will remain anonymous and that all responses will be kept confidential.

## Results

Phase one study

The pilot study was conducted in July 2023 and focused on violence against healthcare workers in the emergency department of RCHSP, Jubail, who had been employed for at least the past six months. Spanning a month, the study utilized a 12-question survey with 35 participants. Of these, a significant majority, 88.6%, were nurses, while physicians comprised 11.4%. Notably, there were no responses from other healthcare worker categories. Demographically, the majority of respondents were male, accounting for 57.1%, and the predominant age group was 35-40 years, representing 42.9% of the participants. Furthermore, a considerable proportion of the respondents, 62.9%, were non-Saudi nationals.

A total of 37.1% of the participants had experienced one to three times verbal violence in the last six months, and 11.4% had experienced physical violence against them or their colleagues. The majority of these incidents, 94.3%, were commented on by patient's families than patients.

At the conclusion of phase one of the study, it was established that nurses are the healthcare workers most frequently subjected to violence. Notably, 94% of these violent incidents were perpetrated not by patients themselves but by patients' relatives, friends, or watchers. Furthermore, 88% of the violence occurred prior to the patient's consultation with a doctor, with 25.7% happening before patient triaging and 62% after triaging but before the doctor's consultation.

The study also revealed that while some staff members do not report violent incidents at all, the rates of formal reporting to security departments and managers are higher than those of informal reporting. In light of these findings, phase two of the study is set to be initiated, focusing on a comparative analysis of the three main hospitals in the region with respect to our participants' experiences.

Phase two study

Sociodemographics

The survey engaged 157 healthcare providers across various specialties from three main hospitals in the Eastern region: Qatif Central Hospital (n=62, 39.5%), Dammam Medical Complex (n=52, 33.1%), and Royal Commission Hospital in Jubail (n=43, 27.4%). A significant majority of the participants, totaling 125 (79.6%), were female.

In terms of age demographics, the largest group comprised those younger than 30 to 35 years old, accounting for 110 participants (70.1%). The majority of participants were of Saudi nationality (n=118, 75.2%). Staff nurses formed the bulk of the respondents, with 110 participants (70.1%) in this category. The highest educational attainment for most was a Bachelor's degree, held by 101 participants (64.3%). The predominant work schedule was 12-hour shifts, reported by 113 staff members (72.0%), and the experience level most represented was between five and 15 years, encompassing 81 participants (51.1%).

Experiencing Verbal Violence for the Last Six Months

The study quantified the frequency of both experiencing and witnessing verbal and physical violence over the last six months. Regarding experiencing verbal violence, 40 participants (25.5%) reported encountering it four to six times, while 32 participants (20.4%) experienced it more than seven times. In the context of physical violence, 29 participants (18.5%) reported one to three incidents.

On the other hand, witnessing verbal violence was reported one to three times by 47 participants (29.9%), four to six times by 43 participants (27.4%), and more than seven times by 44 participants (28.0%). For physical violence, witnessing frequencies were one to three times for 55 participants (35.0%), four to six times for eight participants (5.1%), and more than seven times for seven participants (4.5%), as detailed in Table 1.

**Table 1:** The frequency of the experiencing and witnessing of either verbal of physical in the last six months (n=157)

Question in the questionnaire		N	(%)
In the last 6 months, how frequently have you experienced incidents of verbal violence from patients, their family members, or others in the healthcare setting?	Never	21	(13.4%)
	Rarely (1-3 times)	64	(40.8%)
	Occasionally (4-6 times)	40	(25.5%)
	Frequently (7 or more times)	32	(20.4%)
In the last 6 months, how frequently have you experienced incidents of physical violence from patients, their family members, or others in the healthcare setting?	Never	120	(76.4%)
	Rarely (1-3 times)	29	(18.5%)
	Occasionally (4-6 times)	4	(2.5%)
	Frequently (7 or more times)	4	(2.5%)
In the last 6 months, how frequently have you witnessed incidents of verbal violence directed at your colleagues by patients, their family members, or others in the healthcare setting?	Never	23	(14.6%)
	Rarely (1-3 times)	47	(29.9%)
	Occasionally (4-6 times)	43	(27.4%)
	Frequently (7 or more times)	44	(28%)
In the last 6 months, how frequently have you witnessed incidents of physical violence directed at your colleagues by patients, their family members, or others in the healthcare setting?	Never	87	(55.4%)
	Rarely (1-3 times)	55	(35%)
	Occasionally (4-6 times)	8	(5.1%)
	Frequently (7 or more times)	7	(4.5%)

Causes of Violence

The study's findings indicate that in 56.7% of cases, patients or their companions (82.2%) were identified as the primary perpetrators of violence. Specifically, male patients were reported as the primary cause of verbal or physical violence 65.6% of the time, in contrast to female patients, who were identified as responsible for only 40.8% of incidents.

Regarding the staff's experiences with violence, the results reveal that verbal violence was the most common type, occurring in 82.4% of cases, while physical violence was reported in 63.7% of cases. Notably, nurses were the most frequent victims of violence, accounting for 129 cases (75.8%), followed by non-healthcare staff (n=62, 39.2%), doctors (n=52, 34.1%), and pharmacists (n=20, 12.7%). The majority of violent incidents occurred during the night shift (56.7%), which was significantly higher compared to the morning shift (28%), as detailed in Table 2.

**Table 2:** Frequency of the primary cause of violence

Question in the questionnaire	Strongly agree	Agree	Neutral	Disagree	Strongly disagree
	n (%)				
In your experiences of violence or witnessing it, are patients the primary cause of verbal or physical violence?	26 (16.6%)	63 (40.1%)	48 (30.6%)	18 (11.5%)	2 (1.3%)
In your experiences of violence or witnessing it, are patients' relatives/friends/watchers the primary cause of verbal or physical violence?	65 (41.4%)	64 (40.8%)	23 (14.6%)	4 (2.5%)	1 (0.6%)
In your experiences of violence or witnessing it, is male most commonly committing verbal or physical violence?	40 (25.5%)	63 (40.1%)	45 (28.7%)	8 (5.1%)	1 (0.6%)
In your experiences of violence or witnessing it, is female most commonly committing verbal or physical violence?	18 (11.5%)	46 (29.3%)	68 (43.3%)	20 (12.7%)	5 (3.2%)
In your experiences of violence or witnessing it, is verbal violence the most commonly occurring type of violence?	88 (56.1%)	57 (36.3%)	9 (5.7%)	1 (0.6%)	2 (1.3%)
In your experiences of violence or witnessing it, is physical violence the most commonly occurring type of violence?	6 (3.8%)	15 (9.6%)	36 (22.9%)	86 (54.8%)	14 (8.9%)
In your experiences of violence or witnessing it, are doctors having most incidents of violence against them?	11 (7%)	41 (26.1%)	51 (32.5%)	39 (24.8%)	15 (9.6%)
In your experiences of violence or witnessing it, are nurses having most incidents of violence against them?	78 (49.7%)	41 (26.1%)	27 (17.2%)	9 (5.7%)	2 (1.3%)
In your experiences of violence or witnessing it, are pharmacists having most incidents of violence against them?	4 (2.5%)	16 (10.2%)	43 (27.4%)	65 (41.4%)	29 (18.5%)
In your experiences of violence or witnessing it, are non-healthcare workers having most incidents of violence against them?	3 (1.9%)	24 (15.3%)	38 (24.2%)	59 (37.6%)	33 (21%)
In your experiences of violence or witnessing it, do morning shifts have the most incidents of violence?	8 (5.1%)	36 (22.9%)	67 (42.7%)	39 (24.8%)	7 (4.5%)
In your experiences of violence or witnessing it, do night shifts have the most incidents of violence?	33 (21%)	56 (35.7%)	52 (33.1%)	16 (10.2%)	0 (0.0%)

Reporting a Violence Incidence

In the study, we separately inquired about the likelihood of reporting a violent incident either experienced personally or witnessed against a colleague to various authorities. The findings reveal that 92 participants (58.6%) would likely report such incidents formally to the security department, while 93 participants (59.3%) would opt for a formal report to their immediate manager. Conversely, 83 participants (52.9%) indicated a preference for an informal report to the security department, and 86 participants (54.7%) would choose to report informally to their immediate manager. Notably, 41 participants (26.1%) expressed they would not report the incident to any authority.

Further, to assess the propensity of staff to report verbal or physical violence against colleagues, separate queries were made. The results indicated that 80 participants (51%) would formally report to their immediate manager, and 84 participants (53.5%) would formally report to the security department. Informal reporting preferences were slightly lower, with 76 participants (48.4%) choosing to report to the security department and an equal number (48.4%) to their immediate manager. A significant minority of 50 participants (31.8%) stated they would not report the incident to any authority (see Table 3).

**Table 3:** Frequency of the likelihood of respondent or colleague experiencing verbal or physical violence incident being reported

Question in the questionnaire	Very likely	Likely	Neutral	Unlikely	Very unlikely
	n (%)				
After experiencing verbal or physical violence against you, how likely are you to report the incident formally to the security department?	43 (27.4%)	49 (31.2%)	43 (27.4%)	19 (12.1%)	3 (1.9%)
After experiencing verbal or physical violence against you, how likely are you to report the incident formally to your immediate manager?	51 (32.5%)	42 (26.8%)	38 (24.2%)	23 (14.6%)	3 (1.9%)
After experiencing verbal or physical violence against you, how likely are you to report the incident informally to the security department?	30 (19.1%)	53 (33.8%)	49 (31.2%)	21 (13.4%)	4 (2.5%)
After experiencing verbal or physical violence against you, how likely are you to report the incident informally to your immediate manager?	39 (24.8%)	47 (29.9%)	42 (26.8%)	24 (15.3%)	5 (3.2%)
After experiencing verbal or physical violence against you, how likely are you to not report it to anyone?	11 (7%)	30 (19.1%)	51 (32.5%)	38 (24.2%)	27 (17.2%)
After experiencing verbal or physical violence against a colleague, how likely are you to report the incident formally to your immediate manager?	37 (23.6%)	43 (27.4%)	51 (32.5%)	22 (14%)	4 (2.5%)
After experiencing verbal or physical violence against a colleague, how likely are you to report the incident formally to the security department?	30 (19.1%)	54 (34.4%)	45 (28.7%)	24 (15.3%)	4 (2.5%)
After experiencing verbal or physical violence against a colleague, how likely are you to report the incident informally to the security department?	19 (12.1%)	57 (36.3%)	50 (31.8%)	22 (14%)	9 (5.7%)
After experiencing verbal or physical violence against a colleague, how likely are you to report the incident informally to your immediate manager?	25 (15.9%)	51 (32.5%)	52 (33.1%)	21 (13.4%)	8 (5.1%)
After experiencing verbal or physical violence against a colleague, how likely are you to not report it to anyone?	11 (7%)	39 (24.8%)	48 (30.6)	40 (25.5)	19 (12.1%)

In analyzing the relationship between the length of working shifts and exposure to violence among healthcare professionals, chi-squared tests were employed. The results indicated no statistically significant differences in most aspects of violence exposure. However, an exception was noted in the context of witnessing incidents of physical violence against colleagues perpetrated by patients, their family members, or others within the healthcare setting. This specific scenario demonstrated a statistically significant association with the length of working shifts, as detailed in Table 4.

**Table 4:** Chi-squared test result for the association between the length of the working shift and either being exposed or not exposed to violence among the healthcare staff

Question in the questionnaire		How long is your shift?			Total	Chi-squared value	p-value
		8 hours (n=31)	9.5 hours (n=13)	12 hours (n=113)			
Experienced incidents of verbal violence from patients, their family members, or others	Not exposed (%)	4 (2.5%)	4 (2.5%)	13 (8.3%)	21	3.742	0.15
	Exposed (%)	27 (17.2%)	9 (5.7%)	100 (63.7%)	136		
Experienced incidents of physical violence from patients, their family members, or others	Not exposed (%)	9 (5.7%)	3 (2%)	25 (15.9%)	37	0.646	0.72
	Exposed (%)	22 (14%)	10 (6.4%)	88 (56%)	120		
Witnessed incidents of verbal violence directed at your colleagues by patients, their family members, or others in the healthcare setting	Not witnessed (%)	4 (2.5%)	2 (1.3%)	17 (10.8%)	23	0.095	0.95
	Witnessed (%)	27 (17.2%)	11 (7%)	96 (61.1%)	134		
Witnessed incidents of physical violence directed at your colleagues by patients, their family members, or others in the healthcare setting	Not witnessed (%)	13 (8.3%)	12 (7.6%)	62 (39.5%)	87	9.455	0.00*
	Witnessed (%)	18 (11.5%)	1 (0.6%)	51 (32.5%)	70		

## Discussion

According to the World Health Organization (WHO), violence is the intentional use of physical force or power, whether threatened or used, against oneself, another person, a group, or a community, with consequences or a high likelihood of causing harm, death, mental distress, maldevelopment, or deprivation [3]. Emergency departments are typically dynamic and stressful areas within hospitals, characterized by high workloads, the nature of care, unpredictable events, little control over workflow, high patient load and turnover [14], and extended working hours. This environment may increase the risk of aggression and violence from patients and visitors [5].

Workplace violence (WPV) against healthcare workers in EDs is a significant issue affecting healthcare worker safety and the experiences of patients and families. This study aimed to estimate the prevalence and risks of WPV against healthcare workers in emergency departments in three hospitals in the Eastern Province of Saudi Arabia over the last six months.

Previous studies have documented the prevalence of WPV against healthcare workers in EDs in various locations, such as the United States [1], Turkey [6], Hong Kong [7], and Australia [8], highlighting a high prevalence of violence. In Abha, Saudi Arabia, WPV prevalence was estimated at 58%, with the highest incidence among nurses (63%) [11]. Studies in Egypt and Turkey reported WPV prevalences of 59.7% [15] and 74% [16], respectively.

This study found substantial evidence of WPV, both verbal and physical. One hundred fifty-seven healthcare providers from three main hospitals in the Eastern region participated: Qatif (39.5%), Dammam (33.1%), and Jubail (27.4%). The majority were female (79.6%) and aged younger than 30 to 35 years (70.1%). Most participants were Saudi nationals (75.2%), nurses (70.1%), and had bachelor's degrees (64.3%). The majority worked 12-hour shifts (72.0%), with experience ranging from five to 15 years (51.1%).

The survey revealed that 25.5% experienced verbal violence four to six times and 20.4% more than seven times in the last six months. Physical violence was experienced one to three times by 18.5% of participants. Witnessing violence was also common: 29.9% witnessed verbal violence one to three times, 27.4% witnessed four to six times, and 28% more than seven times in the last six months. Physical violence was witnessed one to three times by 35% of participants. These results reflect a considerable level of WPV in EDs. A majority (82.2%) reported experiencing verbal violence, aligning with findings from Zafar et al. [17].

The study showed that 82.2% of violence incidents were committed by patients' companions and 56.7% by patients, similar to findings from Riyadh [12] and contrasting with a study in China [18]. These results suggest the need for strategies targeting both patients and their companions.

It was noted that males committed violence in 65% of incidents, highlighting the need to consider gender in preventive plans. Nurses were the most affected group (75.8%), followed by doctors (34.1%) and pharmacists (12.7%), reflecting the workflow in EDs.

Associations between working shift lengths and exposure to violence showed no significant differences. However, significant differences were found among professions, with nurses facing the most violence.

Regarding reporting practices, 58.6% reported violence incidents formally to the security department and 59.3% to their immediate manager. However, inconsistencies in reporting practices suggest the need for standardized processes [19].

Limitations

Using non-probability convenience sampling may limit generalizability and introduce response bias. The cross-sectional nature of the study restricts causality inference. Reliance on self-reported data could lead to inaccuracies in violence reporting. Focusing on Eastern Saudi Arabia may not reflect other regions' experiences.

## Conclusions

The study conclusively indicates that workplace violence against healthcare workers is a prevalent issue in emergency departments, with patients and their companions frequently implicated in such events. The data reveals that around 19% of the reported cases constituted physical violence, while verbal abuse accounted for more than 50%. Notably, companions of patients were involved in a majority of these incidents, representing 82.2% of cases. However, the study did not find definitive correlations between the experience of violence and factors such as working hours, the governance structure of the hospital, or awareness of effective reporting systems. Despite this, many incidents were formally reported, underscoring a level of awareness and responsiveness among healthcare workers.

This scenario underscores the need for heightened attention toward identifying the prevalence of all forms of workplace harassment, including physical and verbal abuse, sexual harassment, and bullying by patients and their caregivers. There is a critical need for policy changes at both local and national levels to address and mitigate workplace violence in healthcare settings, thereby ensuring a safer and more secure environment for healthcare professionals.
